# Efficacy and Safety of Runzaozhiyang Lotion for Chronic Hand Eczema: Protocol for a Randomized Controlled Trial

**DOI:** 10.2196/76555

**Published:** 2025-09-24

**Authors:** Rui Zang, Chen-Chen Xu, Bing-Nan Cui, Jia-Ning Bi, Ao-Lin Song, Fan Wei, Jiao Yang

**Affiliations:** 1 Department of Dermatology, Guang’anmen Hospital, China Academy of Chinese Medical Sciences, No. 5, Beixian Pavilion, Xicheng District, Beijing, 100053, China, 86 13301099759

**Keywords:** runzaozhiyang lotion, chronic hand eczema, clinical trial, traditional Chinese medicine, topical medication

## Abstract

**Background:**

Chronic hand eczema (CHE) is a prevalent inflammatory skin disease in clinical practice. Guidelines recommend the use of topical medications, including corticosteroids and calcineurin inhibitors; however, prolonged use of these drugs may result in adverse effects, such as skin atrophy, drug tolerance, and rebound phenomena. Moreover, these medications exhibit limited efficacy in managing chronic hypertrophic hand eczema. Traditional Chinese medicine (TCM) has a long history of treating patients. The Runzaozhiyang lotion (RZZYL) is often used in the Department of Dermatology of Guang'anmen Hospital, China Academy of Chinese Medical Sciences, for these patients, with marked efficacy on symptoms, keratinization, hypertrophy, and pruritus improvements. Nonetheless, there is still no high-quality evidence from evidence-based medicine to prove the effectiveness and safety of CHE treatment.

**Objective:**

This study aimed to examine the efficacy and safety of a classic Chinese herbal prescription, RZZYL, for CHE and to provide a different treatment option for patients with hand eczema who are resistant to conventional Western pharmaceutical treatments.

**Methods:**

This study is a clinical trial characterized as randomized, double-blind, double-dummy, and positive drug-controlled. A total of 122 patients diagnosed with CHE have been randomized and allocated in a 1:1 ratio into 2 distinct groups, with one receiving the intervention and the other serving as the control. Participants in the intervention group will receive RZZYL granules along with a placebo cream of mometasone furoate, whereas the control group will get mometasone furoate cream in combination with a placebo for RZZYL granule. Both groups will be treated with either the active drug or a placebo for 4 weeks. The primary outcome metrics are the Hand Eczema Severity Index (HECSI) and the Numerical Rating Scales (NRS). Secondary outcome metrics consist of the target lesion area score, the Patient-Oriented Eczema Measure (POEM), the Dermatology Life Quality Index (DLQI), and the Investigator Global Assessment (IGA). The outcomes will be assessed at the start of the study and again at 2 and 4 weeks after the treatment period.

**Results:**

The study will be conducted according to the guidelines and regulations approved by the participating institutions. Recruitment is expected to commence in March 2025 and conclude in March 2026. Data collection is anticipated to be completed by June 2026, with the study expected to conclude in August 2026.

**Conclusions:**

This protocol addresses the limitations of previous research and aims to explore the efficacy and safety of RZZYL in treating CHE through a higher-quality clinical study. It seeks to provide additional treatment options for patients with CHE who are resistant to or have poor responses to current therapies.

## Introduction

Eczema is a type of inflammatory disorder that affects the dermis and epidermis. It is caused by both internal and environmental factors. This prevalent clinical condition is categorized into acute, subacute, and chronic eczema according to its progression and clinical features. The hands are a common site for the onset of this condition. Chronic hand eczema is characterized by episodes that occur at least twice a year or persist for more than 3 months during each occurrence. Clinically, chronic hand eczema (CHE) is distinguished by dermal thickening, lichenification, hyperkeratosis, and pruritus [1]. CHE is highly prevalent in the general population, with studies worldwide reporting an incidence rate of 4.0% in the general population and up to 14.5% of individuals experiencing the condition throughout their lifetime [2]. This disorder has become a significant global public health issue in recent years.

At present, there are no medications sanctioned by the US Food and Drug Administration (FDA), specifically for CHE, and a significant unmet demand persists for effective treatments to attain long-term management of CHE [1]. Topical treatment is the conventional method for CHE, and topical corticosteroids (TCS) are the primary suggested therapy. Nevertheless, up to 65% of cases encounter recurrence, skin shrinkage, and other adverse outcomes post treatment, and no evidence indicates that TCS can achieve long-term management of CHE [3]. Calcineurin inhibitors, as an alternative to TCS, can be used for long-term maintenance therapy. These agents help regulate the immune system and reduce inflammation. However, their clinical efficacy in treating skin thickening and lesion-like changes remains suboptimal [4,5]. While some studies have demonstrated that crisaborole (a phosphodiesterase four inhibitor), delgocitinib [6], and ruxolitinib effectively treat hand eczema, large-scale clinical trials are still needed to confirm their efficacy and safety. Furthermore, the high costs associated with these medications impose a significant financial burden on patients [7]. Consequently, current treatment options for CHE in Western medicine remain limited, highlighting an urgent clinical need to identify alternative medications that specifically target the keratotic hyperplasia symptoms associated with CHE.

Traditional Chinese medicine (TCM), as a form of complementary and alternative medicine, has a longstanding history of usage in China for the treatment of hand eczema and remains widely practiced both domestically and internationally [8]. As a unique part of TCM diagnostic and therapeutic techniques, the external treatments of TCM have a long history of success in treating dermatological disorders. Over-the-counter TCM topical preparations, such as Qingpeng Ointment and Longzhu Ointment, are commonly used in China for the treatment of hand eczema. These preparations are adequate for anti-inflammatory and anti-itch therapies. However, Qingpeng Ointment is suitable for acute and subacute eczema, demonstrating limited efficacy for chronic eczema [9,10]. In contrast, Longzhu Ointment is primarily used for treating acne, with no clinical trial evidence supporting its use in treating eczema [11].

Runzaozhiyang lotion (RZZYL) is derived from the *Galla chinensis* ointment developed by Mr Zhu Renkang, the founder of the Department of Dermatology at the China Academy of Chinese Medical Sciences, Guang’anmen Hospital. It combines the traditional *Galla chinensis* ointment formula with the clinical experience of the esteemed Beijing physician, Professor Xu Xian. This formula has established itself as one of the standard prescriptions in the Department of Dermatology at Guang’anmen Hospital, demonstrating apparent clinical efficacy. The primary ingredients of RZZYL comprise *Galla chinensis*, *Tribulus terrestris, Cannabis fructus, Bletilla striata, Cnidium monnieri* (L) Spreng, and *Sophora flavescens* Aiton. Pharmacological research has underscored the diverse therapeutic properties associated with these components (given in Table 1). Our preliminary investigation suggests that this formula exhibits a markedly superior efficacy rate relative to placebo in the treatment of CHE. However, there is a lack of high-quality randomized controlled trials, and the current evidence is insufficient to validate the effectiveness and safety of RZZYL for CHE. Therefore, our study aims to thoroughly evaluate the efficacy and safety of RZZYL for treating CHE through a carefully designed randomized controlled trial.

**Table 1.** Overview of Runzaozhiyang lotion.

Chinese name	Common name	Latin name	Species and family	Proportion (%)	Efficacy
Wubeizi	Chinese Nutgall	*Galla chinensis*	The female aphids of *Aphis gossypii* or *Aphis craccivora* from the Aphididae family of the Hemiptera order, after piercing the tender leaves or petioles of *Rhus chinensis* (or its related species), parasitize them to form galls. These galls can be obtained by baking and drying them.	22.2	Antibacterial and anti-inflammatory effects [12]
Baijili	Caltrop	*Tribulus terrestris*	The mature dried fruits of *Tribulus terrestris*.	22.2	Antibacterial and anti-inflammatory effects [13]
Huomaren	Hemp Fruit	Cannabis fructus	The dried mature fruits of the Moraceae family plant *Cannabis sativa* L.	22.2	Antibacterial, anti-inflammatory, lipoid-regulating, and immunomodulatory properties effects [14]
Baiji	*Bletilla striata*	*Bletilla striata*	The tubers (or bulbs) and underground corms, after being washed, soaked, or blanched, are sliced thinly and then dried in the sun.	11.1	Wound healing, antibacterial, and anti-inflammatory effects [15,16]
Shechuangzi	Common Cnidium Fruit	*Cnidium monnieri* (L) Spreng	The dried mature fruits of the Apiaceae plant *Cnidium monnieri* (L) Cuss.	11.1	Antipruritic, antibacterial, and anti-inflammatory effects [17,18]
Kushen	Light yellow Sophora Root	*Sophora flavescens* Aiton	The dried roots of the leguminous plant *Sophora flavescens* Ait.	11.1	Antipruritic, antioxidant, and anti-inflammatory effects [19,20]

## Methods

### Trial Design

**Figure 1.**
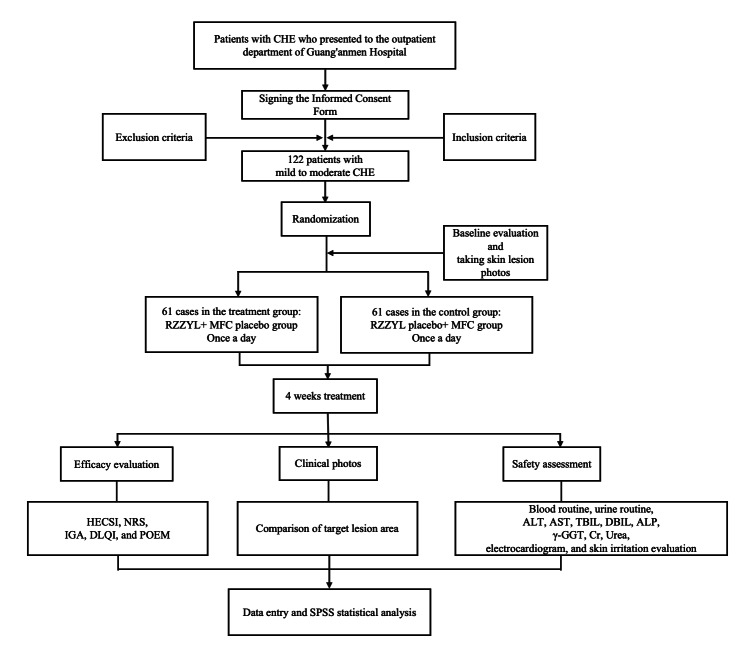
Flow diagram of the randomized double-blind study on the treatment of chronic hand eczema with the Runzaozhiyang Lotion. ALP: alkaline phosphatase; ALT: alanine aminotransferase; AST: aspartate aminotransferase; CHE: chronic hand eczema; Cr: creatinine; DBIL: direct bilirubin; DLQI: dermatology life quality index; γ-GGT: gamma-glutamyl transferase; HECSI: Hand Eczema Severity Index; IGA: Investigator Global Assessment; MFC: mometasone furoate cream; NRS: numerical rating scale; POEM: Patient-Oriented Eczema Measure; RZZYL: Runzaozhiyang lotion; TBIL: total bilirubin.

The trial is randomized, double-blind, double-dummy, and positive drug-controlled. The research will be carried out at Guang’anmen Hospital of the China Academy of Chinese Medical Sciences. Before therapy, patients with CHE have standardized baseline examinations, including medical history, physical examination, specialist assessments, and laboratory tests. Dermatologists will conduct the evaluation scales. The hospital’s laboratory and electrocardiogram departments will test blood, urine, and electrocardiogram. All enrolled patients will be randomly assigned to 2 groups: RZZYL (granules) plus mometasone furoate cream placebo and RZZYL (granules) placebo plus mometasone furoate cream. The treatment method for each patient will be the same within their respective groups. The trial will comply with the World Medical Association Declaration of Helsinki and China’s legislation and recommendations on Good Clinical Practice. Informed consent will be acquired from all participants after a comprehensive explanation by the doctors prior to the intervention. The study design is illustrated in Figure 1.

### Sample Size

This trial aims to objectively assess the efficacy and safety of RZZYL for CHE through a high-quality clinical study design, thereby providing substantial data support for medication development and translation. The trial uses mometasone furoate cream as the positive drug control, categorizing it as a noninferiority trial. According to pertinent literature, the efficacy rate of mometasone furoate cream for treating HE, as assessed by the symptom improvement index using the Nimodipine method, is 53% [21]. Based on previous clinical observations, the efficacy rate for the RZZYL (granules) group is 73.47% [22]. Using the noninferiority study formula (equation 1) [23], with a statistical power (1-β) set at .9, an alpha level (α) of .025, and a noninferiority margin (δ) of 0.095 [24], the values for z_α_ and z_β_ are 1.95996 and 1.28155, respectively, with k (prespecified noninferiority margin) set to 1. The symbol “z” typically represents the critical value in a standard normal distribution, used to calculate the critical region in hypothesis testing. The sample size calculation indicates that the control group requires approximately 53 participants (n_c_). Consequently, the sample size for the experimental group (n_t_) is calculated as k×n_c_=53 participants, leading to a total sample size of 106 participants. Considering an anticipated attrition rate of 15.0% [25], the necessary sample size is adjusted to 61 participants for both the control and experimental groups, resulting in a total required sample size of 122 participants.


(1) $$n_{c}=\frac{\left(Z_{1-\alpha }+Z_{1-\beta }{)}^{2}\cdot [P_{C}(1-P_{C})+P_{T}(1-P_{T})\right]}{(P_{C}-P_{T}-\delta {)}^{2}}$$

### Participants and Recruitment

The inclusion, exclusion, and exit criteria are listed in Textbox 1.

Textbox 1. **Inclusion, exclusion, and exit criteria.**

**Inclusion criteria**

Individuals who satisfy the subsequent criteria will be incorporated into the trial:

- Diagnosis of mild to moderate CHE by the diagnostic criteria established in the Chinese Expert Consensus on the Diagnosis and Treatment of Hand Eczema (2021 Edition) [26] (HECSI score ≤27).
- Diagnosis of chronic eczema classified as blood deficiency and wind-dry type according to the Expert Consensus on the Diagnosis and Treatment of Eczema in Traditional Chinese Medicine (2021 Edition) [27].
- Patients aged 18-70 years (inclusive), both male and female.
- The primary affected region is the hands.
- Signed clinical informed consent and ability to adhere to the treatment protocol.

**Exclusion criteria**

Individuals meeting any of the following criteria will be excluded from participation

- Presence of any systemic or active skin disorders (eg, psoriasis) or conditions that could affect the evaluation of skin lesions, such as scars, tattoos, birthmarks, or other pigmentary skin conditions.
- Existence of specific bacterial, viral, or fungal infections at the site of application that require antibiotic therapy.
- The presence of serious concomitant primary diseases impacting the heart, cerebrovascular system, liver, kidneys, or hematological system, along with individuals experiencing mental health issues.
- Use of systemic corticosteroids or immunosuppressants within the 30 days leading up to treatment; administration of biologics or small molecule medications within the preceding 90 days; or application of topical corticosteroids or retinoids within a week prior to treatment.
- Confirmed allergies to any component of the investigational drug.
- Pregnant or breastfeeding individuals, as well as those planning to conceive in the next 3 months.
- Participation in other clinical trials for pharmaceuticals in the last 3 months.

**Exit criteria**

Patients will be withdrawn from the trial under the following conditions:

- Protocol noncompliance: Missing one or more scheduled follow-up visits without prior notification or valid justification; failure to complete at least 80% of the required clinical assessments (eg, symptom diaries and laboratory tests).
- Safety or medical reasons: Development of a new condition (eg, severe infection and pregnancy) requiring intervention that conflicts with the trial; concurrent use of medications outside the prescribed protocol, particularly those affecting the evaluation of the investigational drug’s efficacy or safety.
- Participant-initiated withdrawal: Voluntary withdrawal via written or verbal request; loss to follow-up (no contact for 14 days or more despite 3 attempted reach-outs via phone or email).
- Other: Death or investigator-determined necessity to terminate the trial (eg, Grade 3 or higher adverse event).

### Concomitant Treatments and Forbidden Drugs

All systemic and topical medications administered to participants during the clinical trial, excluding routine study drugs, will be documented. The reporting period commences from the date of informed consent signing or the randomization date and extends to the final follow-up visit (the date, month, and year of signing will be specified when the informed consent form is signed). Participants must submit their medical history and medication records from the past 3 months to verify medication usage and the date of discontinuation. To eliminate the potential interference from other therapeutic agents, participants are restricted from using systemic corticosteroids, immunosuppressants, biologics, small molecules, or any additional treatments, aside from the standard study medication, from 30 days prior to the informed consent signing or randomization until the final follow-up. Applying topical Chinese patent medicines, corticosteroids, retinoids, calcineurin inhibitors, or other medications is prohibited within 1 week before the final follow-up.

### Recruitment

This research mainly focuses on outpatient individuals from Guang’anmen Hospital, which is connected to the China Academy of Chinese Medical Sciences. This trial is scheduled to conduct a pilot recruitment from March to April 2025 to achieve 5% of the target enrollment. Full patient recruitment is anticipated from May 2025 to March 2026, using advertisements, posters, and physician referrals. The advertisements and posters will provide a brief overview of the trial, outline the inclusion criteria and compensation details, and include the contact information for the researchers. Prior to enrollment, all participants will receive a thorough explanation of the trial procedures, the objectives of the research, possible adverse effects, and expected benefits. During the screening phase, each individual’s eligibility for participation will be evaluated, and they will be informed of their right to withdraw from the study. Those who decide to participate in the trial will be required to complete 2 informed consent documents: one for their records and one to be kept by the research team.

To enhance patient retention, the following measures will be implemented:

1. Follow-up reminders: SMS text messaging and WeChat (Tencent) reminders will be dispatched 48 hours and 24 hours prior to the appointment.
2. Incentive system: A participant subsidy of 200 RMB (approximately US $27.64) will be provided upon the completion of all follow-up visits.
3. Convenience optimization: Weekend follow-up slots will be made available.

### Randomization, Concealment, and Blinding

This study will use the SAS (SAS Institute) PROC PLAN process statement to generate random numbers to determine the seed number and fragment length. The treatment tasks corresponding to run numbers 001‐120 will be documented. To ensure allocation concealment, the randomization process will be overseen by an independent statistician who is not involved in the study [28]. Eligible participants will be randomly assigned to either the treatment or control groups in a 1:1 ratio. The random numbers will be placed in a closed, opaque envelope, secured in our center. To ensure objectivity, operators will not be aware of any evaluations by efficacy evaluators and vice versa. Only the operators will have access to the random numbers prior to the commencement of the treatment. Further, they are not going to take part in the evaluation. To maintain strict allocation concealment, the system shall log metadata, such as time of randomization, operator’s identity, and IP address. The quality control will regularly check these logs for any violations or deviations. In order to blind the participants, both groups will receive the same package of drug and placebo, with a similar treatment protocol. The placebo will add artificial coloring to ensure size, color, shape, taste, odor, and packaging are all identical to the active drug. Unblinding will occur only after the completion of case collection. However, an emergency unblinding procedure may be initiated if a serious adverse event or urgent medical treatment is necessary during the study.

Furthermore, the quality control team will conduct anonymous surveys at 3 critical stages, including when the trial reaches (1) 50% completion, (2) just before its conclusion, and (3) before unblinding. These surveys will evaluate whether participants, clinical researchers, and efficacy evaluators are aware of the group allocations. The data collected from these surveys will be subjected to statistical analysis to mitigate the risks of performance and detection biases, thereby enhancing the study’s credibility.

### Intervention

Patients will initially dilute the granules (either the moisturizing and anti-itch wash or the placebo wash) in 1500‐2000 mL of hot water during the treatment period. When the medication cools to around 37°C, instruct the patient to fully immerse both hands in the solution for 15 minutes. After this, the medication will be rinsed off. Following the wash, an external cream (either mometasone furoate cream or placebo cream) will be applied to the affected area using the fingertip unit method, where 1 fingertip unit is sufficient to cover the entire hand of an adult (both front and back). The cream will be applied evenly in a thin layer to cover the lesions fully, followed by gentle circular massaging to enhance absorption. This application will occur once daily. Daily care will be administered throughout the day using Xiehe Vitamin E Cream (Product Standard number SuG Cosmetics Network Backup 2019002615). Clinical researchers will distribute medications biweekly to mitigate the risk of early dropout associated with a single 4-week supply. During the week 4 follow-up, both used and unused wash and cream will be collected, and panoramic photographs of the medication bags and tubes (including the participant ID label) will be taken and archived. A follow-up phone check will be conducted for medications that are not returned within 48 hours to confirm usage frequency and document the reasons for nonreturn. The sensitivity analysis will include participants with adherence rates below 80% or exceeding 120%. Furthermore, all medications used in the study will only be distributed after passing quality control testing.

### Outcome Measures

**Figure 2.**
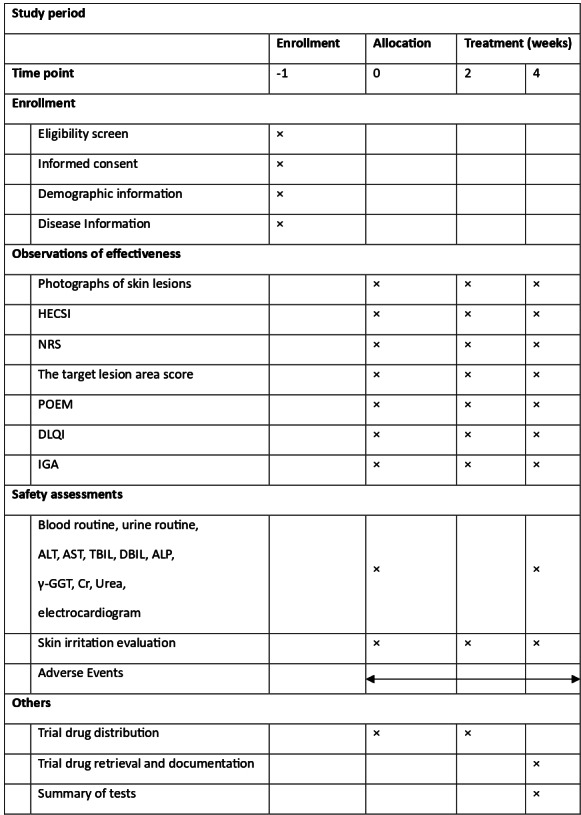
Timeline for trial enrollment, intervention, and assessments. ALP: alkaline phosphatase; ALT: alanine aminotransferase; AST: aspartate aminotransferase; Cr: creatinine; DBIL: direct bilirubin; DLQI: Dermatology Life Quality Index; HECSI: Hand Eczema Severity Index; IGA: investigator global assessment; NRS: numerical rating scale; POEM: Patient-Oriented Eczema Measure; TBIL: total bilirubin; γ-GGT: gamma-Glutamyl transferase.

Figure 2 shows the study timeline. As detailed below, all outcome measurements, along with drug administration and collection, will be documented from baseline to the conclusion of treatment.

### Primary Outcomes

#### Hand Eczema Severity Index

Hand Eczema Severity Index (HECSI) serves as a global standard for assessing the severity of skin lesions, facilitating a quantitative description of the clinical manifestations of hand eczema and enabling the monitoring of treatment outcomes [29]. The HECSI scoring system categorizes each hand into 5 distinct regions, such as fingertip, fingers (excluding the fingertips), palm, back of the hand, and wrist. Skin lesions are categorized as erythema (E), papules (I), scales (S), and fissures (F). Areas are assessed based on the intensity of skin lesions, with points allocated for no lesions (0), mild alterations (1), moderate alterations (2), and severe alterations (3). The total lesion area of both hands is assessed as follows: no involvement is assigned a score of 0. For involvement between 1% and 25%, the score is 1. Involvement between 26% and 50% is scored as 2, while involvement between 51% and 75% corresponds to a score of 3, and 76% to 100% corresponds to a score of 4. The total HECSI score is derived from the aggregate of scores for each site, specifically the sum of the lesion scores, which is then multiplied by the lesion area score. The score categorizes hand eczema into 3 classifications, including mild (0‐11 points), moderate (12‐27 points), and severe (≥28 points).

#### The Numerical Rating Scale

The numerical rating scale (NRS) is a horizontal line segment akin to the Visual Analog Scale, generally anchored at one end as “no itching” and at the other as “extremely itchy” or “the worst itching ever experienced,” with the end points marked as “0” and “10,” respectively. A score of 0 indicates the absence of itching while a score of 10 signifies the highest level of itching possible. A higher score indicates increased severity of itching. Patients report a numerical value reflecting the average intensity of itching experienced over the preceding 24 hours or the past week. Itching severity is categorized as no itching (score 0), mild itching (0<score <4), moderate itching (4≤ score <7), severe itching (7≤ score <9), and very severe itching (score ≥9).

### Secondary Outcomes

#### The Target Lesion Area Score

This experiment focuses exclusively on hand lesions; thus, a single representative lesion is chosen as the target lesion. The lesion area will be assessed prior to treatment and after the trial. The percentage reduction in area will be determined from the measurements and used for comparative analysis.

#### Patient-Oriented Eczema Measure

The Patient-Oriented Eczema Measure (POEM) scoring scale has 7 questions that assess the severity of hand eczema symptoms experienced last week. Each question is scored on the basis of frequency or severity in the range of 0 to 4 points. The total score is the sum of all individual scores. Scores can range from 0 to 28 points. A more severe score denotes a greater severity of eczema symptoms and a more substantial impact on the patient’s quality of life.

#### Dermatology Life Quality Index

The Dermatology Life Quality Index (DLQI) measures the quality of life of patients with skin diseases. It measures both the symptoms and the impact of the skin disease on the patient’s everyday activities. This scale has a total of 10 questions regarding the patient’s life in the last week. These are emotional, professional, and social experiences. The scoring for each question is done on a scale of 0-3; the higher the score, the greater the impact of the condition on the quality of life of the person. The total score is the total of all individual responses. For this score, the higher the score, the worse the patient’s quality of life.

#### Investigator Global Assessment

Researchers and clinicians use the Investigator Global Assessment (IGA) score to gauge a patient’s skin condition. The score is often used in clinical trials for eczema and other skin diseases. Rating the severity of a disease depends on the characteristics of skin lesions, such as redness, exudation, and thickness. The dermatological revised scoring system works on a 5-point scale, ranging from 0 to 4, where higher scores indicate more severe skin conditions.

### Safety Assessment

Safety indicators will be evaluated through the subsequent assessments:

1. General physical examination: The patient’s general condition and basic vital signs (body temperature, blood pressure, pulse, and heart rate) will be monitored and recorded both prior to and following enrollment. The participants’ health status will be assessed to determine eligibility for the trial and monitor health changes throughout the trial.
2. Laboratory assessments: At allocation and after 4 weeks of treatment, the assessments that will be conducted are blood routine, urine routine, alanine aminotransferase, aspartate aminotransferase, gamma-glutamyl transferase, total bilirubin, direct bilirubin, alkaline phosphatase, creatinine, urea, and electrocardiogram.
3. Evaluation of skin irritation: Skin erythema, blisters, dryness, scaling, pigmentation changes, tightness, and itching will be assessed and documented at allocation and after a 4-week treatment period.

During each visit to the trial, adverse events were recorded in the participant’s pathological report form. If required, all serious adverse events and unanticipated events should be immediately reported to the Guang’anmen Hospital Ethics Committee and relevant authorities. If the injury assessment confirms that the injury caused by the research is related to the trial, the treatment and compensation costs will be borne by Guang’anmen Hospital of the China Academy of Chinese Medical Sciences. If there is a significant change in the research protocol related to the participant or a substantial increase in the related, it will be essential to resecure his informed consent. In addition, a committee will track participant safety and integrity of the study, called the Data and Safety Monitoring Committee (DSMC). The principal investigator will regularly assess all reported adverse events. Based on necessity, the principal investigator may call for a meeting with the research team to evaluate the risks and benefits. Scheduled interim analyses will be performed to monitor the study’s ongoing safety and amend the study protocol if necessary.

### Oversight and Monitoring

### Data Collection and Management

Original documents will be prepared for each participant randomly assigned to the study by the researchers. All pertinent information will be documented in the case report forms and archived in Microsoft Excel spreadsheets. Under the direction of the principal investigator, an internal data monitoring team will oversee and audit the clinical data to maintain data integrity and quality. The case report forms will be reviewed regularly or intermittently, and timely feedback will be provided to the researchers if any issues are identified. All participant data will be maintained strictly confidential and not accessible to the public.

### Data Analyses

The results of this trial will be analyzed from three perspectives:

1. Full Analysis Set (Intention-to-Treat Analysis): This set includes all randomized participants, with data analyzed according to the principle of “randomization equals analysis.” Missing data will be addressed using multiple imputation methods. Predictive mean matching will be used for continuous variables while logistic regression imputation will be used for categorical variables.
2. Per Protocol Set: This set comprises participants who meet specific criteria; that is, they must have completed at least 80% of the treatment regimen (ie, a minimum of 22 days of medication within 4 weeks), have not used prohibited study drugs, and possess complete data for the primary end point (HECSI score).
3. Safety Set: This set includes all participants who received at least 1 dose of the study medication.

The variable processing and statistical methods for this trial are outlined as follows: (1) continuous variable data that adhere to a normal distribution will be presented as mean (SD). Independent sample *t* tests (2-tailed approach) will facilitate between-group comparisons while repeated measures ANOVA will be applied for comparisons across multiple time points (eg, baseline, during treatment, and post treatment); (2) data that do not conform to a normal distribution will be expressed as median (IQR), with between-group comparisons executed using the Mann-Whitney *U* test; (3) categorical data will be described in terms of proportions or percentages, with between-group comparisons conducted using chi-square tests or Fisher exact test, depending on the final sample size. (4) All data processing and chart generation will be performed using R 4.0 software (R Project for Statistical Computing). Statistical tests will be 2-sided, with a significance level at *P*<.05.

### Quality Control

Prior to the commencement of the trial, all participating researchers will receive standardized training on diagnostic interviews to ensure familiarity with the management process and to clarify the implementation procedures. During the trial, 2 clinical physicians will evaluate CHE. The allocation will be fixed, and migration between groups will not be permitted. The reason for a participant’s withdrawal from the study will be documented if they discontinue treatment.

### Ethical Considerations

#### Ethics Approval

Ethics committee, Guang’anmen Hospital China Academy Of Chinese Medical Sciences, China.

#### Ethics and Dissemination

The trial will be carried out in line with the World Medical Association’s Declaration of Helsinki and the legislation and guidelines on Good Clinical Practice established in China. This protocol has been approved by the Ethics Committee of Guang’anmen Hospital, China Academy of Chinese Medical Sciences (2024‐262-KY), and registered on the International Traditional Medicine Clinical Trial Registry Platform (ITMCTR2025000287). Researchers must thoroughly inform participants regarding the study’s purpose, procedures, potential risks, and compensation. Participants will complete 2 informed consent forms: one for their records and the other for the researchers, to be included in the clinical research documentation for future reference.

To express gratitude to participants for their investment of time and transportation costs, and by the ethical committee’s guidance on reasonable compensation (based on local standards for volunteer participation in research and minimum wage rates), participants will receive a subsidy of 200 RMB (approximately US $27.64) upon the completion of the entire treatment and follow-up. This compensation adheres to ethical guidelines for reimbursement, aiming to avoid undue inducement while ensuring fairness. Participants’ privacy and confidentiality will be rigorously protected throughout the study. When the study results are published, participants’ names will be replaced with a unique serial number and the initials of their names to ensure that personal information is not disclosed. Data handling will comply with applicable data protection regulations, and access to personal information will be restricted to authorized researchers.

## Results

Recruitment is scheduled to commence on March 1, 2025. The initial month will be a pilot phase, targeting an enrollment of 4-7 patients. Subsequently, it is projected that 8-12 participants will be recruited each month. The recruitment process is anticipated to conclude by March 2026, with the results published in the winter of 2026. By July 1, 2025, a total of 40 patients have been recruited. Data analysis and report writing are expected to be finalized by August 2026.

## Discussion

### Strengths and limitations

One limitation is that participants may exaggerate the severity of CHE due to the subjectivity involved in completing self-assessment questionnaires. In addition, the study is carried out in a single hospital, which may limit the applicability of its results. Despite these constraints, the outcomes of this research will contribute new evidence for RZZYL from a meticulously designed trial. These results will promote the advancement of complementary and alternative therapies within TCM for chronic health issues while improving patients’ quality of life.

### Future Directions

RZZYL can potentially reduce inflammation, combat bacterial infections, and facilitate the recovery of skin lesions, thus providing an alternative treatment option for patients with clinically resistant or recurrent chronic hand eczema and enhancing their quality of life. Following the publication of our research findings, we will further promote the RZZYL treatment regimen through formulation development, academic conferences, and training sessions. In addition, we will disseminate the academic results of this project nationwide through academic exchanges, paper publications, and participant reports, thereby facilitating the development, national promotion, and application of the research outcomes.

### Conclusion

This study protocol describes a randomized, double-blind, double-dummy, positive drug-controlled trial to evaluate RZZYL for treating chronic hand eczema (CHE). The trial will assess the efficacy and safety of RZZYL, using rigorous quality control and precise methodologies to ensure high validity and reliability. Detailed methods for allocation concealment, recruitment, randomization, and data collection have been thoroughly outlined. Objective measures such as scale scoring, lesion area, and laboratory tests will be used to evaluate the treatment's effectiveness and safety. The results may provide insights into RZZYL's potential as an alternative treatment to the current TCS for clinically resistant or recurrent CHE.

## Abbreviations

CHE: chronic hand eczema
DLQI: Dermatology Life Quality Index
DSMC: Data and Safety Monitoring Committee
FDA: US Food and Drug Administration
HECSI: Hand Eczema Severity Index
IGA: Investigator Global Assessment
NRS: numerical rating scale
PDE-4: phosphodiesterase four inhibitor
POEM: Patient-Oriented Eczema Measure
RZZYL: Runzaozhiyang lotion
TCM: traditional Chinese medicine
TCS: topical corticosteroids
